# The preoperative predictors for subsequent degeneration in L5-S1 disc after long fusion arthrodesis terminating at L5 in patients with adult scoliosis: focus on spinopelvic parameters

**DOI:** 10.1186/s13018-018-0987-7

**Published:** 2018-11-13

**Authors:** Changzhi Yan, Xianda Gao, Yadong Sun, Zhen Dong, Yong Shen

**Affiliations:** Department of Spine Surgery, The Third Hospital of Hebei Medical University, No. 139 Ziqiang Road, Shijiazhuang, Hebei People’s Republic of China

**Keywords:** Adult scoliosis, Preoperative predictors, Long fusions, Disc degeneration, Spinopelvic parameters

## Abstract

**Background:**

The subsequent L5-S1 disc degeneration associated with long fusion arthrodesis terminating at L5 in patients with adult scoliosis has been a common concern. However, few studies paid attention to its preoperative predictors, especially in spinopelvic parameters. The purpose of the present study was to clarify the preoperative predictors of subsequent L5-S1 disc degeneration after long fusion arthrodesis terminating at L5 in patients with adult scoliosis on spinopelvic parameters.

**Methods:**

In this retrospective study, we enrolled 67 patients with adult scoliosis, and the patients were divided into disc degeneration group (DD) and no disc degeneration group (NDD), based on the presence or absence of subsequent L5-S1 disc degeneration. The status of L5-S1 disc was evaluated by a modified version of radiographic classification. Characteristics and spinopelvic parameters of preoperative patients were collected as potential predictors for subsequent lumbosacral disc degeneration after long fusion arthrodesis terminating at L5 in patients with adult scoliosis. Multivariate logistic regression analysis and the receiver operating characteristic curve were used to identify the preoperative predictors, with an adjusted odds ratio (OR) and 95% confidence intervals (CI).

**Results:**

Thirty-six patients (53.73%) with subsequent L5-S1 disc degeneration were divided into group DD (preoperative score 0.81 ± 0.57, last follow-up score 1.83 ± 0.60, *P <  0.001*), and the other 31 patients were divided into group NDD (preoperative and last follow-up score 0.87 ± 0.49). There was no statistical difference in preoperative score (*P* = 0.583) of lumbosacral disc between two groups; however, significant statistical difference showed in last follow-up score (*P* <  0.001). Multivariate logistic regression identified three preoperative predictors: pelvic incidence (PI) (*P* = 0.018), sagittal vertical axis (SVA) (*P* = 0.024), and sacrum-femoral distance (SFD) (*P =* 0.023). PI < 48.5° (OR = 0.911, 95% CI = 0.843–0.984), SVA > 4.43 cm (OR = 1.308, 95% CI = 1.036–1.649), and SFD > 5.65 cm (OR = 1.337, 95% CI = 1.041–1.718) showed satisfied accuracy for predicting subsequent L5-S1 disc degeneration.

**Conclusion:**

The prevalence of the subsequent L5-S1 disc degeneration after long fusion arthrodesis terminating at L5 in patients with adult scoliosis was 57.3% (36 of 67 patients). PI < 48.5°, SVA > 4.43 cm, and SFD > 5.65 cm were preoperative predictors for the subsequent L5-S1 disc degeneration. More attention should be paid to prevent the L5-S1 disc from degeneration when these preoperative predictors exist, especially with two or more.

## Background

Adult scoliosis, a spinal deformity with Cobb angle greater than 10° after skeletal maturation, occurs in two types: idiopathic adolescent scoliosis in adulthood and de novo scoliosis [1]. Although long fusion arthrodesis for adult scoliosis revealed satisfactory clinical outcomes including spinal reconstruction and symptom relief of obstinacy low-back pain, radicular pain, and intermittent claudication, the distal fixed vertebrae terminating at L5 or sacrum has remained controversial [2–4].

The distal fusion segment extending to the sacrum is undisputed for patients with adult scoliosis with presented severe lumbosacral disc degeneration, instability of lumbosacral segment, L5 spondylolysis, and nerve compression at L5-S1 needing decompression [4]. Long fusion arthrodesis terminating at L5 is a better choice without existing pathological changes mentioned above, and it showed obvious advantages of minor surgery, smaller infection probability, shorter anesthesia time, and lower incidence of pseudarthrosis [2, 4]. However, long fusion arthrodesis terminating at L5 usually leads to a potential risk for subsequent degeneration in L5-S1 disc. With the subsequent L5-S1 disc degeneration, low back pain, nerve compression, and decompensation of balance in the coronal plane and sagittal plane occurred [2–6]. Due to the complications, patients after long fusion arthrodesis terminating at L5 required revision surgery whose distal fixed vertebrae should extend to S1 or even to the iliac bone [2, 3, 6]. Previous studies have paid attention to complications, risk factors, radiographic parameters, surgical outcomes, and revision surgery, which are caused by subsequent degeneration in L5-S1 disc [2, 4, 6–9]. Nevertheless, just a few studies discussed the preoperative predictors of subsequent degeneration in L5-S1 disc after the long fusion arthrodesis terminating at L5 in patients with adult scoliosis, although it was beneficial for surgical strategy.

Therefore, the purpose of our study was to clarify the preoperative predictors of subsequent degeneration in L5-S1 disc after long fusion arthrodesis terminating at L5 in patients with adult scoliosis and to provide evidence for surgical strategy. Besides, spinopelvic parameters have been addressed in the current study because of their important role in subsequent lumbosacral disc degeneration.

## Materials and methods

### Patients

This retrospective study was approved by the Institutional Ethics Board of the Third Hospital of Hebei Medical University. The retrospective study included a consecutive series of 67 patients with adult scoliosis who underwent surgical treatment at our institution from May 2004 and March 2016. The inclusion criteria were as follows: (1) presence of adult scoliosis with Cobb angle greater than 10°, (2) posterior-only surgical instrumented procedure terminating at L5, (3) no history of any spinal surgery, (4) fixed segments greater than or equal to four, and (5) follow-up period more than 2 years. The exclusion criteria were as follows: (1) lack of completed clinical data, (2) disability of lower limb, and (3) tumor or inflammation involving the spine. Finally, 49 women and 18 men with mean age at surgery of 59.24 years (range, 39–77 years) were reviewed in the study. Forty-one patients (61.19%) had de novo scoliosis in adulthood and 26 patients (38.81%) had a history of idiopathic adolescent scoliosis without treatment. The mean follow-up period was 4.85 years (range, 2–9 years). Clinical data, including age, gender, body mass index (BMI), number of instrumented vertebrae, and spinopelvic parameters, were collected as potential predictors for subsequent lumbosacral disc degeneration.

### Clinical and radiographic analysis

**Fig. 1:**
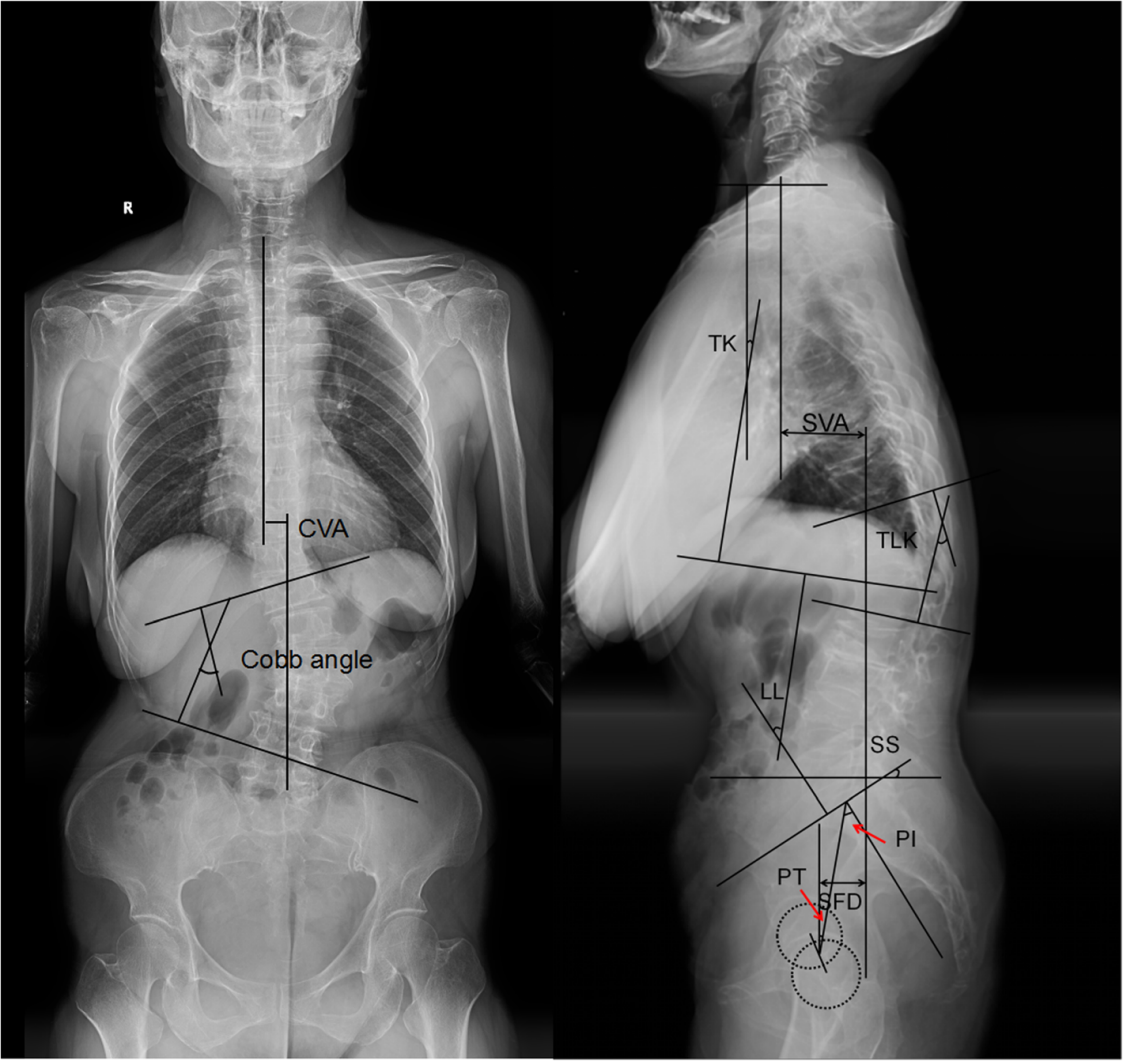
Illustration of radiographic measurements taken from anteroposterior and lateral radiographs of the entire spine

Spinopelvic parameters were measured on anteroposterior and lateral radiographs of the entire spine (Fig. 1). The sagittal vertical axis (SVA), coronal vertical axis (CVA), Cobb angle, pelvic incidence (PI), sacral slope (SS), pelvic tilt (PT), lumbar lordosis (LL), thoracic kyphosis (TK), thoracolumbar kyphosis (TLK), L5 oblique angle, and sacrum-femoral distance (SFD) were collected in this study. SVA equal to or more than 5 cm was considered as sagittal imbalance, which was defined as the distance from the C7 plumb line to the posterior sacral prominence [7, 8]. CVA was defined as the horizontal distance between the central sacral vertical line and C7 plumb line. The Cobb angle was defined as the angle between the superior endplate of the upper end vertebrae and inferior endplate of the lower end vertebrae. TK was defined as the angle between the superior endplate of T1 and L1 [10]. TLK was defined as the angle between the superior endplate of T11 and the inferior endplate of L1 [10]. PI was defined as the angle between the perpendicular of the sacral plate and the line connecting the middle point of the sacral plate and the middle point of the bilateral femoral head center [11, 12]. SS corresponds to the angle between the sacral plate and the horizontal plane [13]. PT corresponds to the angle between the vertical and the line connecting the midpoint of the sacral plate to the middle point of the bilateral femoral head center [11, 12, 14]. LL was defined as the angle between the superior endplate of L1 and the superior endplate of S1 [10]. L5 oblique angle was defined as the angle between the superior endplate of L5 and the interiliac crest line [15]. SFD was defined as the horizontal distance between the posterior sacral prominence and the middle point of the bilateral femoral head center [10]. The status of L5-S1 disc was evaluated according to a modified version of radiographic classification described by Weiner et al. [3, 16], which included four grades (0 = no degeneration, 1 = mild degeneration, 2 = moderate degeneration, 3 = sever degeneration) and five parts of assessment criteria: loss of disc height, osteophyte formation, endplate eburnation, vertebral listhesis, and gas in the disc space. The anteroposterior and lateral radiographs of the entire spine were taken preoperatively and at the last follow-up visit. All relevant spinopelvic parameters were measured three times by the same spinal surgeon, and the average value was used for analysis.

### Statistical analysis

The data was analyzed by statistical software, the SPSS 21.0 software (IBM, Inc., Chicago, IL). Results are presented as the mean ± standard deviation (SD). Quantitative data including age, BMI, degenerative degree, and spinopelvic parameters was analyzed by independent *t* test; if the data was not in accord with normal distribution or homoscedasticity, Mann-Whitney *U* test would be used. Categorical data including gender and number of instrumented vertebrae was analyzed by chi-square test. The statistical significance was set as *P* value less than 0.05. The potential predictors which showed *P* <  0.05 in univariate regression analysis were analyzed by multivariate logistic regression analysis with adjusted odds ratio (OR) and 95% confidence intervals (CI). PI, SVA, and SFD were entered into the logistic regression again to identify the correlation between subsequent disc degeneration and the predictors after adjusting for potential confounding factors. The sensitivity and specificity in PI, SVA, and SFD were evaluated by the receiver operating characteristic curve, and, at the same time, cutoff value in the three parameters was obtained to achieve good predicting.

## Results

The status of L5-S1 disc was evaluated by a modified version of radiographic classification (Table 1). The preoperative X-rays showed 16 patients in grade 0, 46 patients in grade 1, and five patients in grade 2. At the last follow-up visit, the X-rays showed seven patients in grade 0, 30 patients in grade 1, 27 patients in grade 2, and three patients in grade 3. Subsequent disc degeneration was defined when the last follow-up disc grade was greater than preoperative disc grade. Finally, 36 patients (53.73%) were divided into group disc degeneration (group DD) with last follow-up average score 1.83 ± 0.60; the other 31 patients were divided into the group no disc degeneration (group NDD) with last follow-up average score 0.87 ± 0.49 (*P* <  0.001). However, no significant statistical difference was found in preoperative average score between the two groups (group DD 0.81 ± 0.57, group NDD 0.87 ± 0.49) (*P* = 0.583). The number of instrumented vertebrae was four in three patients, five in four patients, six in 11 patients, seven in eight patients, eight in 28 patients, nine in six patients, ten in four patients, and 11 in three patients. There was no significant statistical difference in instrumented vertebrae number between group DD (7.56 ± 1.64) and group NDD (7.52 ± 1.54) (*P* = 0.927). Significant statistical difference was not found in average follow-up period (*P* = 0.377), BMI (*P* = 0.207), and gender (*P* = 0.355) (Table 2).

**Table 1:** Radiographic scoring system for osteoarthritis of the lumbosacral spine intervertebral disc

Score	Characteristic
0	No degeneration, defined by normal disc height, no spur formation, no eburnation, no listhesis, no gas
1	Mild degeneration, defined by 25% disc space narrowing, small spur formation, minimal eburnation, no listhesis, and no gas
2	Moderate degeneration, defined by 25 to 75% disc space narrowing, moderate spur formation, moderate eburnation, listhesis 3 mm, and no gas
3	Advanced degeneration, defined by 75% disc space narrowing, arge spur formation, marked eburnation, listhesis 5 mm, gas present

**Table 2:** Comparison of patient characteristic between DD and NDD groups

Variables	Group DD (*n* = 36)	Group NDD (*n* = 31)	*P*
Age (year)	59.6 ± 8.3	58.8 ± 8.9	0.706^a^
Gender			0.355^c^
Male	8	10	
Female	28	21	
BMI (kg/m^2^)	22.95 ± 3.43	23.93 ± 2.61	0.207^a^
Instrumented vertebrae number			0.927^c^
4–6	9	9	
7–9	23	19	
> 9	4	3	
Follow-up period (year)	4.70 ± 1.67	5.02 ± 1.77	0.377^b^
Pelvic incidence (°)	48.53 ± 7.84	54.03 ± 7.89	0.006^a^
Pelvic tilt (°)	22. 53 ± 4.33	24.58 ± 4.27	0.058^a^
Sacral slope (°)	26.19 ± 6.38	29.13 ± 5.47	0.051^a^
Cobb angle (°)	46.11 ± 6.03	44.95 ± 7.92	0.402^a^
Sagittal vertical axis (cm)	6.65 ± 3.09	4.12 ± 2.67	0.001^a^
Coronal vertical axis (cm)	2.76 ± 1.22	2.35 ± 1.49	0.235^a^
Lumbar lordosis (°)	37.43 ± 8.70	35.84 ± 9.43	0.482^a^
Thoracic kyphosis (°)	37.23 ± 9.59	35.51 ± 8.86	0.457^a^
Thoracolumbar kyphosis (°)	11.53 ± 4.81	9.96 ± 4.03	0.162^a^
L5 oblique angle (°)	8.26 ± 2.38	7.51 ± 2.61	0.291^a^
Sacrum-femoral distance (cm)	6.76 ± 2.56	4.43 ± 2.13	< 0.001^b^
Preoperative radiographic score	0.81 ± 0.57	0.87 ± 0.49	0.583^b^
Radiographic score at last follow-up	1.83 ± 0.60	0.87 ± 0.49	< 0.001^b^

The data in the table are presented as the mean ± SD. *P* < 0.05, significant correlation

^a^Independent *t* test

^b^Mann-Whitney *U* test

^c^Chi-square test

**Fig. 2:**
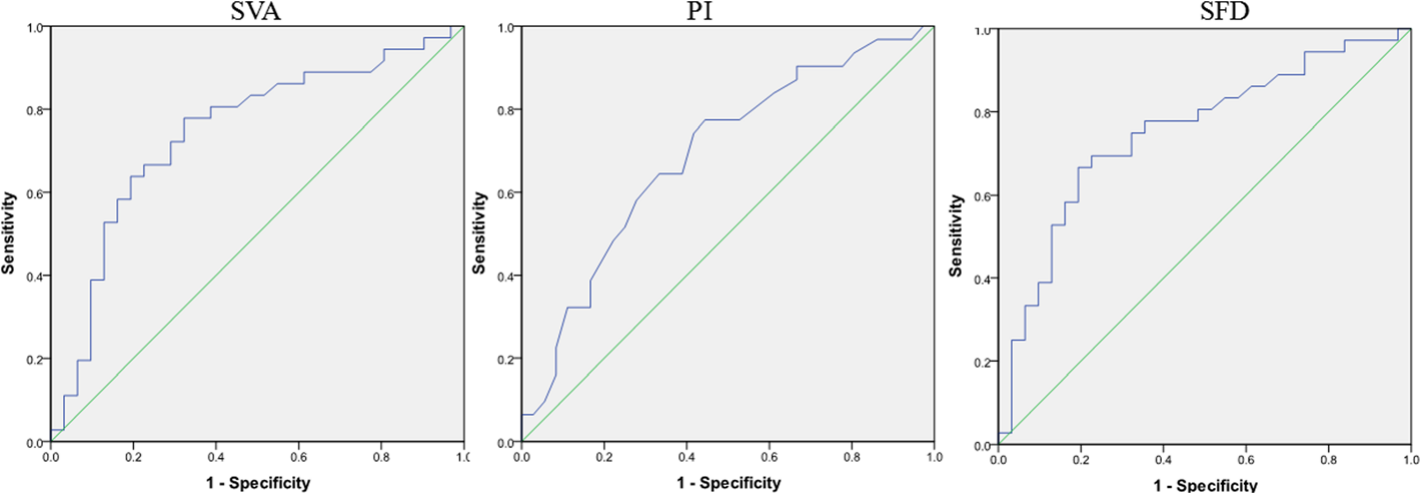
Receiver operating characteristic curves of preoperative predictors (PI, SVA, SFD)

The relevant spinopelvic parameters and univariate analysis were summarized in Table 2. Comparing group DD and group NDD, PI (48.53 ± 7.84 vs 54.03 ± 7.89, *P* = 0.006), SVA (6.65 ± 3.09 vs 4.12 ± 2.67, *P* = 0.001) and SFD (6.76 ± 2.56 vs 4.43 ± 2.13, *P* < 0.001) showed significant difference. In multivariate logistic regression, PI (OR = 0.911, 95% CI = 0.843–0.984, *P* = 0.018), SVA (OR = 1.308, 95% CI = 1.036–1.649, *P* = 0.024), and SFD (OR = 1.337, 95% CI = 1.041–1.718, *P* = 0.023) were identified to be predictors for subsequent L5-S1 disc degeneration (Table 3). The satisfied accuracy for predicting subsequent L5-S1 disc degeneration in PI (area under the curve 0.690, *P* = 0.008), SVA (area under the curve 0.743, *P* < 0.001), and SFD (area under the curve 0.750, *P* = 0.001) were shown in receiver operating characteristic curve analysis (Fig. 2; Table 4). According to the maximum Youden index, the cutoff value was calculated as 48.5° in PI, 4.43 cm in SVA, and 5.65 cm in SFD (Table 4). The presence of two or three predictors (PI < 48.5°, SVA > 4.43 cm, and SFD > 5.65 cm) were significantly associated with subsequent L5-S1 disc degeneration (*P* = 0.024) (Table 5).

**Table 3:** Predictors for the subsequent L5-S1 disc degeneration: multiple logistic regression analysis

Variable	Adjusted odds radio	95% confidence interval	*P*
Pelvic incidence	0.911	0.843–0.984	0.018
Sagittal vertical axis	1.308	1.036–1.649	0.024
Sacrum-femoral distance	1.337	1.041–1.718	0.023

**Table 4:** Sensitivity, specificity, AUC, and cutoff of predictors

Variable	Sensitivity	Specificity	AUC	Cutoff	*P*
Pelvic incidence	0.774	0.556	0.690	48.5	0.008
Sagittal vertical axis	0.778	0.677	0.743	4.43	< 0.001
Sacrum-femoral distance	0.667	0.806	0.750	5.65	0.001

**Table 5:** Differences in the incidence of subsequent L5-S1 disc degeneration in patients with 0, 1, or ≥ 2 predictors

Variable	Adjusted odds ratio	95% confidence interval	*P*
0 predictor	1		
1 predictor	0.533	0.192–1.477	0.226
≥ 2 predictors	3.167	1.164–8.619	0.024

## Discussion

Adult scoliosis shows to be increasingly common in the aging society. Patients with adult scoliosis hankeringly seek the treatment of doctors, hoping their clinical symptoms get remission. When outcomes of conservative treatment are poor, surgical treatment is a better choice to relieve the symptoms. Nowadays, the surgical treatment of adult scoliosis is widely concerned by spinal surgeons [2–4, 6–9]. Sardar et al. [9] published a retrospective study to contrast the outcomes of long fusion arthrodesis terminating at L5 and extension to the sacrum, showing no statistical significance in both groups; the same result was found in the study of Edwards et al. [4]. Nevertheless, choosing L5 or S1 as the distal fixed vertebrae was still controversial because of different incidence of complications [4, 17–19]. However, Eck et al. [2] held an opposite viewpoint, and his studied showed the incidence of complications in fixed to S1, fixed to L4 and fixed to L5 was no statistical difference, agreed by Kleinstueck et al. [20]. Long fusion arthrodesis terminating at L5 showed theoretical advantages of smaller surgery, smaller infection probability, and preserved lumbosacral motion segment; however, the disadvantage was potential subsequent L5-S1 disc degeneration [2–4, 21].

With the subsequent L5-S1 disc degeneration, low back pain, nerve compression in L5 or S1 segment, and decompensation of balance in the coronal plane and sagittal plane occurred [2, 3, 5]. The severe disc degeneration was often related with revision surgery. Edwards et al. [4] reported that 61% (18 in 27) of patients after long fusion arthrodesis terminating at L5 developed into subsequent L5-S1 disc degeneration, and revision surgery was performed in four patients because of severe disc degeneration. Polly et al. [21] reported a patient who underwent long fusion arthrodesis terminating at L5 who received a revision surgery with extension of the fixed to the iliac bone, due to the severe subsequent L5-S1 disc degeneration. In our study, at the last follow-up, subsequent L5-S1 disc degeneration involved 36 of 67 (53.73%) patients with average follow-up period of 4.85 years (range 2–9 years). We revealed three preoperative predictors for the subsequent L5-S1 disc degeneration by multiple regression analysis, which were PI < 48.5° (OR = 0.911, 95% CI = 0.843–0.984), SVA > 4.43 cm (OR = 1.308, 95% CI = 1.036–1.649), and SFD > 5.65 cm (OR = 1.337, 95% CI = 1.041–1.718).

The preoperative SVA > 4.43 cm was identified as a preoperative predictor for the subsequent L5-S1 disc degeneration. Many previous studies proved that the subsequent L5-S1 disc degeneration was highly correlated with sagittal imbalance [7, 8, 10, 22–24]. Kim et al. [22] confirmed that a high preoperative SVA is a risk factor for disc degeneration. Brown et al. [15] demonstrated that preoperative imbalance was correlated with distal disc degeneration. Matsumoto et al. [24] found that preoperative sagittal imbalance was significantly associated with adjacent-segment disease. All of their findings are similar to the result demonstrated in our study. However, Cho et al. [23] drew a conclusion that preoperative sagittal imbalance was not a risk factor for subsequent L5-S1 disc degeneration. On the contrary, they also reported that 8 of 11 (73%) patients with sagittal imbalance developed into subsequent disc degeneration, and they suggested patients with minimal lumbosacral disc degeneration and sagittal imbalance to fuse to S1 because of the high incidence of subsequent disc degeneration. Kuhns et al. [6] proved that the length of the fusion was a risk factor for advancing in L5-S1 disc degeneration; at the same time, they reported that 28% of the patients with instrumented vertebrae ≤ 10 and 72% of the patients with instrumented vertebrae > 10 advanced in L5-S1 disc degeneration. In our study, the number of instrumented vertebrae showed no statistical difference (*P* = 0.927). It may be caused by the reason that the number of patients with instrumented vertebrae > 10 (three patients with fusion from T7 to L5) in our study was limited. Unexpectedly, all the three patients developed into subsequent L5-S1 disc degeneration. The relationship between number of instrumented vertebrae and subsequent L5-S1 disc degeneration should be further studied.

PI, an anatomical parameter, being not affected by lumbar degeneration diseases after the end of bone growth, has been mentioned in previous studies and played a significant role in disc degeneration [10–12, 14, 22, 25]. Yang et al. [10], in a comparative study, compared 60 patients weathered lumbar disc disease with 110 normal volunteers and came to a conclusion that patients with a low PI had a significantly higher risk of disc degeneration. Barrey et al. [26] reported that patients with a mean PI 48.3° had a higher risk of disc degeneration than the patients with a mean PI 52° in the young group. Xu et al. reported 284 patients with lumbar disc degenerative disease and concluded that patients with PI ≤ 50° were more likely to have degeneration at L4/5 and L5/S1 discs [27]. Nevertheless, a retrospective article found that PI and lumbar disc herniation were no significant correlation in young Chinese patients [28]. In the present study, there is a correlation between PI < 43.5° and subsequent disc degeneration. The low PI, symbolizing a more vertical sacrum, implies a flat spine. With a flat spine, the stress of body concentrates in L5-S1 disc and accelerates its degeneration. Moreover, in this study, the SFD was identified as a predictor for the subsequent L5-S1 disc degeneration. The SDF, a pelvic parameter, is used to evaluate the globally balanced state in the sagittal alignment. Yang et al. [10] concluded that a low PI was significantly associated with disc degeneration, accompanied with a high SFD at the same time, and it suggested that a low PI means a high SFD. They thought that a high SFD signifies a more flat spine, and it leads to more stress in the terminal disc. However, the further and clear explanation about the two parameters was not given by them.

Several limitations in the current study needed to be pointed out. First, the study was limited by its retrospective nature. Second, follow-up period in the study was medium-term. However, medium-term follow-up was not enough to evaluate the status of disc degeneration because the process was gradual. Third, the radiographic classification of the disc is divided into only four grades, so a more detailed classification method would be proposed and applied in this study. What is more, the number of patients with adult scoliosis after long fusion arthrodesis terminating at L5 in this study was small. Thus, further multicenter studies with a large sample would be preformed to clarify the correlation between the preoperative predictors and the subsequent L5-S1 disc degeneration.

## Conclusion

The prevalence of the subsequent L5-S1 disc degeneration in this study was 57.3% (36 of 67 patients). The PI < 48.5°, SVA > 4.43 cm, and SFD > 5.65 cm were identified as the preoperative predictors for the subsequent L5-S1 disc degeneration. Spine surgeons should pay more attention in choosing the surgical strategy when the preoperative predictors exist in patients, especially with two or more.

## Abbreviations

CI: Confidence intervals
CVA: Coronal vertical axis
DD: Disc degeneration
LL: Lumbar lordosis
NDD: No disc degeneration
OR: Odds ratio
PI: Pelvic incidence
PT: Pelvic tilt
SD: Standard deviation
SFD: Sacrum-femoral distance
SS: Sacral slope
SVA: Sagittal vertical axis
TK: Thoracic kyphosis
TLK: Thoracolumbar kyphosis
